# Diversity and distribution of Orchidaceae in one of the world's most threatened plant hotspots (Madagascar)

**DOI:** 10.3897/BDJ.11.e106223

**Published:** 2023-09-19

**Authors:** Vincent Droissart, Simon Verlynde, Brigitte Ramandimbisoa, Lalao Andriamahefarivo, Tariq Stévart

**Affiliations:** 1 AMAP Lab, Univ. Montpellier, IRD, CNRS, CIRAD, INRAE, Montpellier, France AMAP Lab, Univ. Montpellier, IRD, CNRS, CIRAD, INRAE Montpellier France; 2 Plant Systematics and Ecology Laboratory, Higher Teachers’ Training College, University of Yaoundé I, P.O. Box 047, Yaoundé, Cameroon Plant Systematics and Ecology Laboratory, Higher Teachers’ Training College, University of Yaoundé I, P.O. Box 047 Yaoundé Cameroon; 3 Herbarium et Bibliothèque de Botanique africaine, CP 169, Université Libre de Bruxelles, Av. F. Roosevelt 50, B-1050, Brussels, Belgium Herbarium et Bibliothèque de Botanique africaine, CP 169, Université Libre de Bruxelles, Av. F. Roosevelt 50, B-1050 Brussels Belgium; 4 Missouri Botanical Garden, Africa & Madagascar Department, P.O. Box 299, St. Louis, Missouri 63166-0299, United States of America Missouri Botanical Garden, Africa & Madagascar Department, P.O. Box 299 St. Louis, Missouri 63166-0299 United States of America; 5 Cullman Program for Molecular Systematics, New York Botanical Garden, Bronx, New York 10458-5126, United States of America Cullman Program for Molecular Systematics, New York Botanical Garden, Bronx New York 10458-5126 United States of America; 6 PhD Program in Biology, Graduate Center, City University of New York, 365 5th Ave., New York, NY 10016, United States of America PhD Program in Biology, Graduate Center, City University of New York, 365 5th Ave. New York, NY 10016 United States of America; 7 Botanic Garden Meise, Domein van Bouchout, Nieuwelaan 38, B-1860 Meise, Belgium Botanic Garden Meise, Domein van Bouchout, Nieuwelaan 38 B-1860 Meise Belgium

**Keywords:** biodiversity hotspot, Malagasy orchids, plant database, sampling gaps, tropical Flora

## Abstract

Introduction. In recent decades, Madagascar has become one of the most important plant hotspots in the world. The country's remaining forests and vegetation are disappearing at an alarming rate, while dozens of new species are discovered each year. Amongst the plant families that have long been studied, Orchidaceae appear as one of the most charismatic, diverse and of high conservation concern. Based on a reviewed, comprehensive herbarium dataset, we have compiled a curated checklist of all orchid species occurring in Madagascar. Based on this complete dataset, we then compiled sampling effort, species diversity distribution and some general statistics on their ecology and IUCN conservation status.

Methods. We compiled and standardised a global dataset using five public databases as the main data sources, supplemented by the most recent publications. The database contains ~ 10,000 geolocated records collected between 1816 and 2021. We used GIS software and rarefaction methods to examine sampling and diversity patterns.

Results. According to our dataset, there are currently 913 orchid species collected in Madagascar, of which 759 orchid species (83.1%) are endemic. Doubling the sampling effort could lead to the discovery of around 100 more species, bringing the total estimated number of orchid species in Madagascar to between 986 and 1048. About one-third (297 species) of all orchid species are known only by type specimens (189 species) or have not been collected in Madagascar for more than 50 years (214 species). Although the raw data show that the Andasibe-Moramanga area would have the highest orchid species concentration, our analysis of the data, adjusted for bias, shows that the centres of orchid diversity in Madagascar are in the Tsaratanàna Strict Nature Reserve and the Ranomafana National Park. Life-form statistics show that 55.0% of orchid species are strict epiphytes. The main flowering period of orchids in Madagascar is between November and March. To date, 84% of the 226 Malagasy orchid species listed in the IUCN Red List are threatened with extinction (CR, EN or VU).

Conclusion. Despite geographically uneven coverage, the biodiversity of Malagasy orchids appears to be already well documented. We provide maps corrected for sampling bias that indicate priority areas for future surveys. Upcoming efforts should also focus on rediscovery and conservation of rare and/or threatened species and ensure that the protected area network is well aligned with the distribution of priority species for conservation. Finally, the conservation status of 75% of the orchid species found in Madagascar is not yet known and the inclusion of these species must be a top priority in the coming years.

## Introduction

Oceanic islands generally harbour a rich endemic flora and Madagascar, the fourth largest island in the world, does not deviate from this rule (Whittaker and Fernández-Palacios 2007). The recently-published *Catalogue of the Vascular Plants of Madagascar* (Callmander et al. 2011, Madagascar Catalogue 2022) confirms the presence of more than 11,500 plant species on the island, of which 80-85% are endemic. This high rate of endemism, combined with the strong human impact compared to mainland regions (Kier et al. 2009), has significant implications for the conservation of the global biodiversity including the highly-diversified Malagasy flora (Antonelli et al. 2022).

Madagascar has experienced extensive deforestation in the last decade and 44% of the natural forest area has been lost in the period 1953–2014 (Vieilledent et al. 2018). Anthropogenic climate change will continue to threaten Madagascar’s exceptional biodiversity. Due to the combination of high endemism and high deforestation rates, Humphreys et al. (2019) identify Madagascar as one of the seven regions in the world with the highest rate of plant species extinction (i.e. 19 extinct species). These previous studies call for better identification of gaps in plant coverage to find rare or unknown species before they disappear.

To date, there have been few studies on the spatial distribution and diversity of plants in Madagascar (but see, for example, Palms: Rakotoarinivo et al. (2014); Sarcolaenaceae: Soulebeau et al. (2016); Pandanaceae: Callmander et al. (2007)) and none focuses on Orchidaceae. In fact, Orchidaceae is the largest family of flowering plants in Madagascar (Madagascar Catalogue 2022) and is known worldwide as flagship for habitat preservation and biodiversity conservation. With approximately 1000 species described to date, orchids make up about 10% of Malagasy flora and up to 85% of them are currently considered endemic (Hermans and Rajaovelona 2022). The orchid flora of Madagascar has been studied by scientists and naturalists for about two centuries (see Hermans and Rajaovelona (2022), Hermans et al. (2007), Hillerman and Holst (1986) for a complete history of Malagasy orchids), but several new species are still being discovered and described each year. Conversely, Roberts and Jarić (2020) have shown that 11 orchid species may be extinct in Madagascar, although the current IUCN Red List (IUCN 2022) has not assessed any extinct orchid species in the island. As Malagasy orchids exhibit a wide range of life forms (i.e. terrestrial, lithophytic or epiphytic) and distribution types (from microendemic to widespread species), we advocate they may be an appropriate test group for evaluating the island’s current botanical sampling coverage.

In this study, we compiled a database of all herbarium records of Madagascar’s largest plant family, the Orchidaceae, to: i) analyse sampling and diversity patterns of orchids in Madagascar, accounting for sampling biases and ii) provide scientists and conservationists with an updated checklist of orchid species collected in Madagascar along with summary statistics on their ecology and conservation. In particular, we addressed the following three important questions: i) how many orchid species have been collected in Madagascar to date and to what extent are these species well known and/or collected? ii) how many species remain to be potentially discovered and iii) what should future plant surveys focus on to increase the likelihood of finding rare or unknown orchid species?

## Material and methods

### Records database and checklist compilation

The main goal of this work is to provide scientists and conservationists with a state-of-the-art dataset and key figures on the diversity and distribution of orchid species in Madagascar. To estimate the total number of orchid species in Madagascar, we compiled a global dataset using five public databases as main data sources: the Missouri Botanical Garden’s Tropicos database (http://www.tropicos.org/, last excerpted 10 March 2021), the GBIF database (GBIF 2019), the MNHN’s vascular plant database (https://science.mnhn.fr/institution/mnhn/collection/p/item/search), the BM database (https://data.nhm.ac.uk/search) and the Kew Herbcat database (http://apps.kew.org/herbcat/). We also reviewed the literature to add new or missing records (Hermans and Cribb 2014, Hermans et al. 2021a, Hermans et al. 2021b, Hermans et al. 2021c, Hermans et al. 2021d, Hermans et al. 2020a, Hermans et al. 2020b, Hermans et al. 2020c, Hermans et al. 2017, Hermans et al. 2021e, Hermans 2020a, Hermans 2020b, Hermans and Cribb 2021). We then used recent literature (e.g. Chase et al. (2020), Chase et al. (2021)) or online databases (IPNI 2022, Madagascar Catalogue 2022, WCVP 2022) to standardise taxonomic names. Our dataset contains specimens from the following 22 herbaria (acronyms after Thiers 2018, continuously updated): AMES, B, BM, BRLU, CNARP, G, HEID, HUNT, K, MO, NM, NU, NY, OS, P, SZU, TAN, TEF, UPS, US, W, WAG.

To avoid taxonomic conflicts, we restricted the identification of specimens to the species level, although some specimens were identified as infraspecific taxa (subspecies or varieties). In addition, because identification of Malagasy orchids can be difficult, we preferred not to use direct observations or photographs without herbarium vouchers (e.g. iNaturalist, https://www.inaturalist.org/ or Pl@ntnet, https://plantnet.org/) to avoid dubious or/and unverifiable data. All occurrences in our dataset are, therefore, supported by specimens deposited in public herbaria.

### Richness estimates and sampling bias

Geographic coordinates of recent collections were recorded using a global positioning system or assigned retrospectively using a gazetteer of botanical collecting localities (e.g. http://legacy.tropicos.org/Project/Madagascar) for old specimens. One should note that locality data for some of the used databases (especially P and K) are sometimes incomplete and this will affect the results of spatial analysis. In order to minimise these biases, we avoided performing fine scale analyses and applied data resampling methods (see below).

Sampling intensity and species richness were calculated using fixed grid cell sizes of 0.5° x 0.5° and 1° x 1°, providing an appropriate balance between precision and detail. Rarefaction methods were used to calculate an unbiased estimate of species diversity per grid cell, Hurlbert’s effective number of species (Ek) found in fixed-size k subsamples (Hardy 2010, Dauby and Hardy 2012). For comparison with raw species richness, we have calculated Ek for k = 10 (i.e. for grid cells in which at least 10 herbarium specimens were collected) and for k = 100. We do not considered Ek values when the number of specimens in a grid cell is equal to the number of species collected in the same grid cell. Richness estimates and sampling completeness were assessed with sample-based rarefaction curves using the R package iNEXT (Hsieh et al. 2016). We used the iNEXT package to calculate the seamless rarefaction (interpolation) and extrapolation (prediction) sampling curves and the associated 95% confidence intervals of individual-based abundance data. All maps were prepared with ArcMap 10.8.1 (ESRI 2020).

### Ecology and IUCN conservation status

Life forms of orchid species were classified into three different growth form types (epiphyte, lithophyte and terrestrial) and were mainly taken from Govaerts et al. (2022) and supplemented by literature or ecological descriptions on specimen labels. Conservation status, when published, was taken from the IUCN Red List Portal (IUCN 2022).

## Results

### Malagasy orchid checklist and database

A total of 913 orchid species, representing 53 genera, are reported from Madagascar (Suppl. material 1). Of these, 759 species (83.1%) are endemic. We compiled a database of 13473 specimens identified to species (Suppl. material 2), representing 11093 unique herbarium records (i.e. excluding the duplicates collected from the same living individuals in shadehouse collections), of which 10300 are georeferenced (890 species).

**Figure 1.**
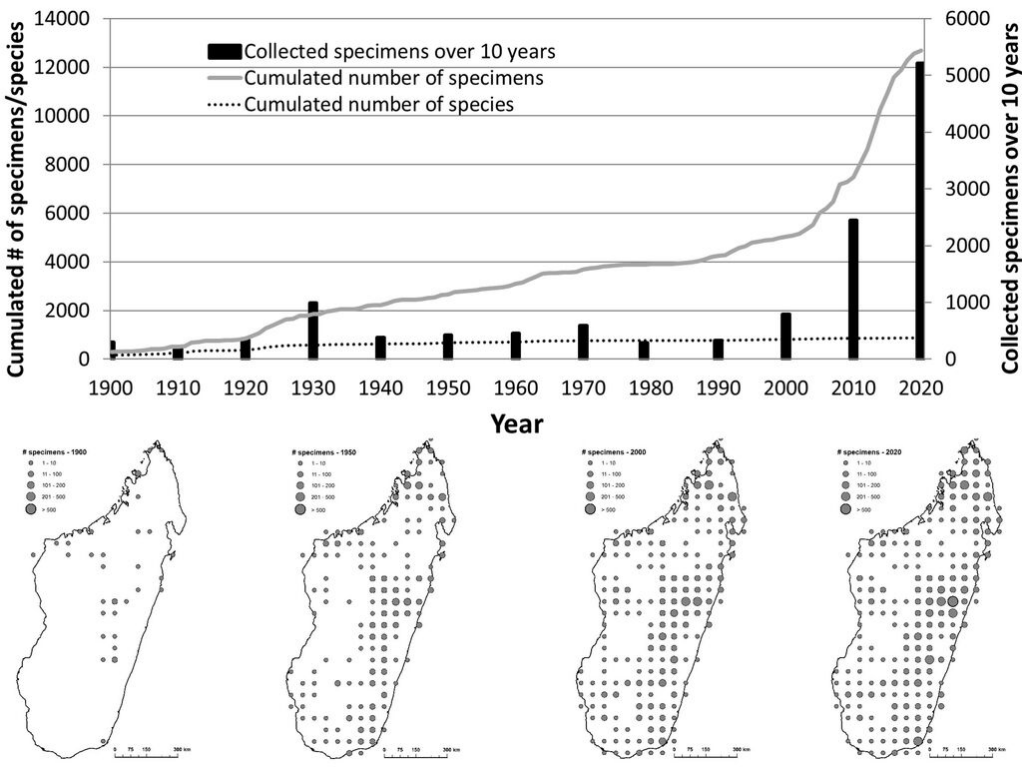
Temporal distribution of orchid’s collections across Madagascar. Maps present the number of botanical collections made within 0.5° grid cells on different dates (1900, 1950, 2000, 2020). The upper figure is based on dated herbarium records (12693 records), while lower maps are based on dated and georeferenced herbarium records (9824 records).

In Madagascar, the first orchid specimens were collected in 1816 (three specimens were collected by Louis-Marie Aubert du Petit-Thouars) and about 60% of specimens were collected after 2000 (Fig. 1). Most specimens were collected by the Ambatovy/MBG teams (1305 unique herbarium specimens collected between 2010 and 2019) and by Henri Perrier de la Bâthie (1134 unique herbarium specimens collected between 1896 and 1953). Our database also shows that 214 species have not been collected for over 50 years and that 189 species are known only from type specimens (Suppl. material 1), which together represent about one third of the Malagasy orchid flora (297 species).

### Sample coverage and species distribution

**Figure 2.**
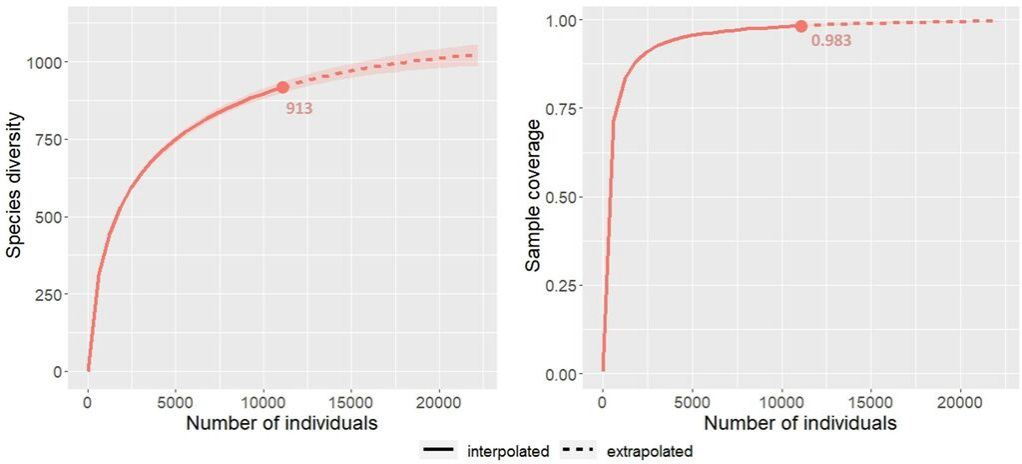
Occurrence-based accumulation curves and sample coverage for Malagasy orchid species diversity. The numbers below rarefaction curves indicate the observed species richness (left) and sample completeness (right). The shaded area represents the upper-lower 95% confidence interval.

The current sample coverage for Malagasy orchid species diversity was strikingly high (98.3%), suggesting that the family is relatively well known in Madagascar (Fig. 2) . From the sample accumulation curves, we can estimate that a doubling of the current sampling effort will result in the discovery of a hundred species and that the total number of orchid species in Madagascar could be estimated between 986 and 1048 species.

**Figure 3.**
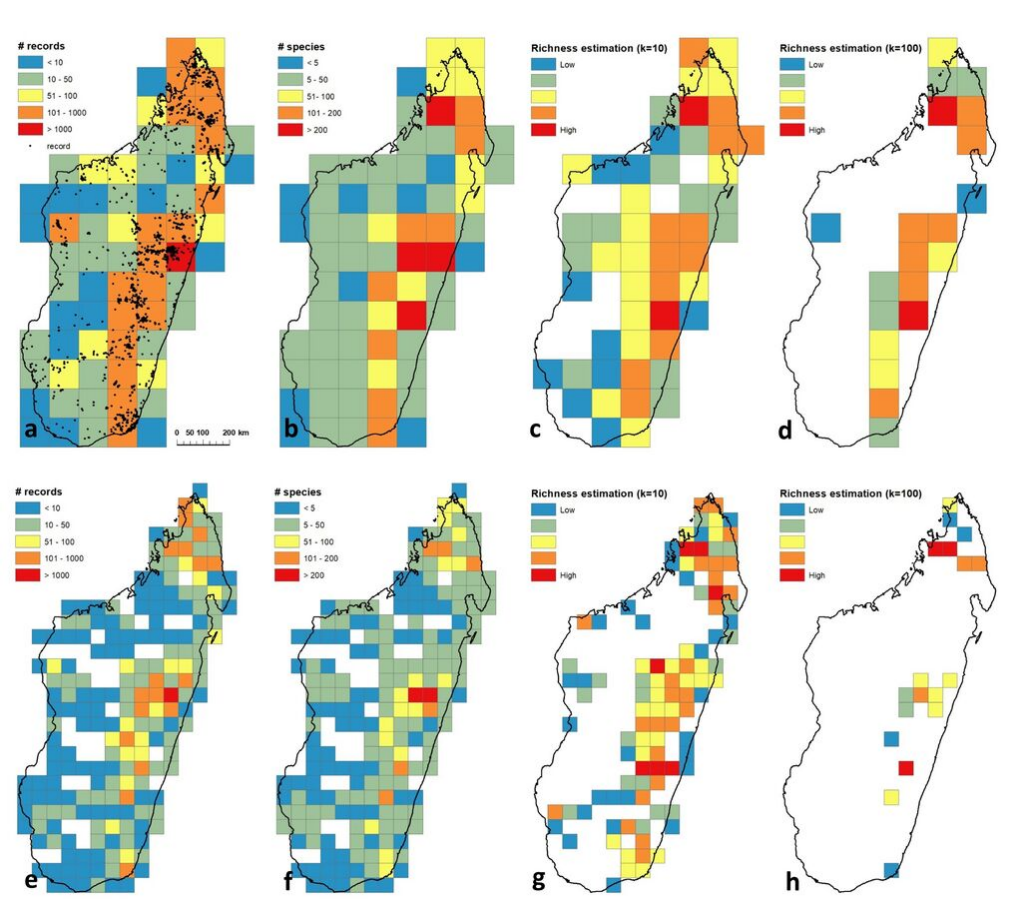
Distribution of orchid records and species richness across Madagascar, using fixed grid cell sizes of 1° x 1° (a-d) and 0.5° x 0.5° (e-h); a and e, number of herbarium records (samples) collected per grid-cells; b and f, number of species collected per grid-cells; c and g, unbiased estimate of species diversity per grid cell, Hurlbert’s effective number of species (Ek) found in grid-cells that contain at least 10 herbarium records (Ek = 10); d and h, unbiased estimate of species diversity per grid cell, Hurlbert’s effective number of species (Ek) found in grid-cells that contain at least 100 herbarium records (Ek = 100).

The large majority of specimens and species known to date have been collected in the moist forest of eastern Madagascar (Fig. 3). In 2012, a large collection programme was designed yielding thousands of new specimens and allowing the discovery of a large number of new species that have yet to be described. The raw data (Fig. 3a,b,e,f) show that the greatest local species richness of Malagasy orchids is found in the Andasibe-Moramanga area. With more than 1000 records per square grid, this area also has the highest sampling intensity. Contrastingly, our analysis of bias-adjusted data shows that the centres of richness of orchids in Madagascar are instead located in the Tsaratanàna Strict Nature Reserve and the Ranomafana National Park (Fig. 3d,h).

### Ecology and conservation

**Figure 4.**
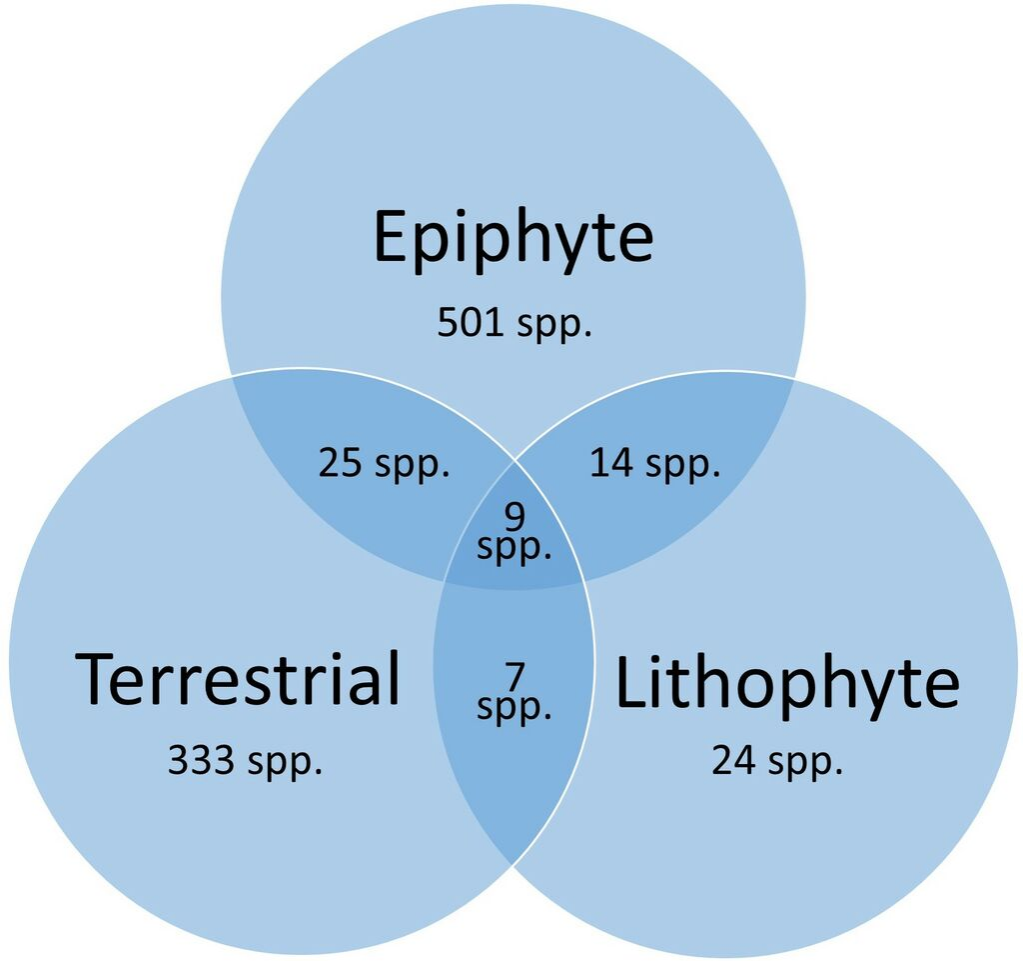
Venn diagram depicting life forms repartition amongst Malagasy orchids (913 species). Different growth form types (epiphyte, lithophyte and terrestrial) were mainly taken from Govaerts et al. (2022), supplemented by literature or ecological descriptions on specimen labels.

Statistics on life forms (Fig. 4) show that 54.9% of the 913 orchid species recorded for Madagascar are strict epiphytes while 36.5% are terrestrial species, 2.6% are lithophytes and 6.0% share at least two life forms.

**Figure 5.**
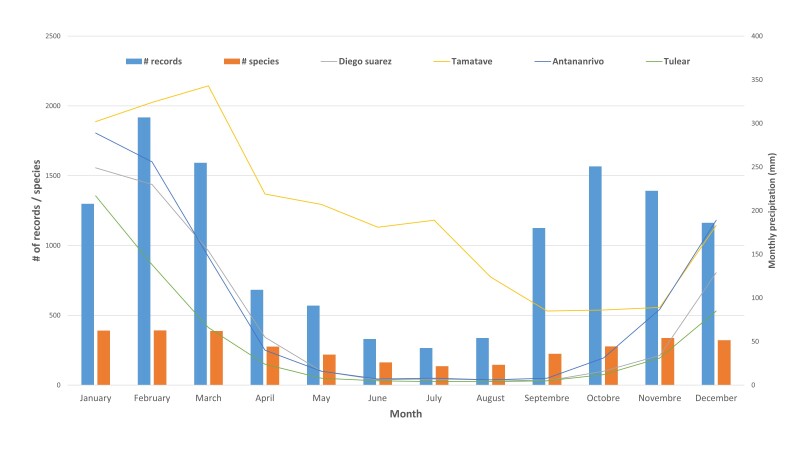
Flowering patterns for orchids in Madagascar. The blue and orange bars indicate the number of samples and the number of species collected with flowers per month, respectively. Monthly precipitation data (lines) were collected between 1999 and 2019 (source: https://climate-data.org/).

The main flowering season of orchids (Fig. 5) in Madagascar is between November and March and about 330 species may flower during these five months. These flowering peaks are congruent with the rainy season in different parts of Madagascar.

**Figure 6.**
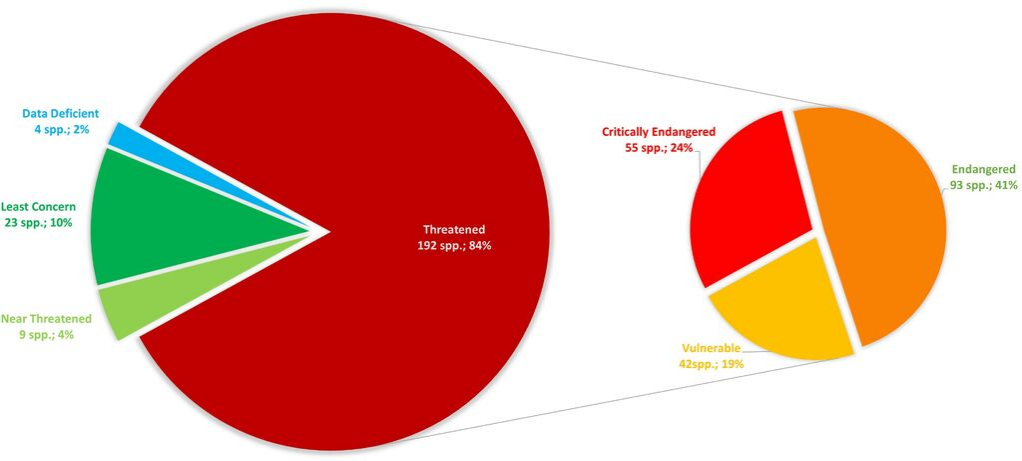
IUCN Red List Categories of the 226 Malagasy orchids assessed to date and published on the IUCN Red List Portal (https://www.iucnredlist.org/).

To date, the extinction risk of 226 Malagasy orchid species has been assessed using the IUCN Red List Categories and Criteria (Fig. 6). Of these fully assessed species, 84% are threatened with extinction: 55 species are classified as Critically Endangered (CR), 93 species as Endangered (EN) and 42 species as Vulnerable (VU).

## Discussion and conclusions

Our database and checklist analyses, as well as recent literature (e.g. Hermans and Rajaovelona (2022)), indicates that Madagascar hosts about 1000 orchid species, confirming their status as the most diverse family in the country, surpassing the Rubiaceae (806 species) and the Fabaceae (603 species), according to the Madagascar Catalogue (2022). The high level of endemism (~ 83%) is consistent with that of the global Malagasy flora, recently estimated at 82% by Antonelli et al. (2022) and once again highlights the importance of this family for conservation of Madagascar’s plant biodiversity.

Of particular significance for the diversity analysis presented here are the potential error margins in the identification of orchid specimens. Indeed, our dataset and subsequent analysis incorporated some herbarium specimen identification as they were originally recorded. Based on our expertise (but also Mackay-Smith and Roberts (2019)), it is important to note that such identifications can carry a substantial margin of error, with higher discrepancies observed for more intricate genera (e.g. *Liparis* Rich. or *Polystachya* Hook.). This highlights the pressing requirement for a thorough reassessment of several genera in Malagasy orchids. Such taxonomic revisions could also serve as a constructive approach to enhance the accuracy of the statistics presented in our study, as well as to help general knowledge and conservation of the rich Malagasy orchid flora.

Many orchid collections in Madagascar are concentrated near roads and within the established nature reserves and parks. Consequently, significant portions of the country, notably remote parts such as the eastern rainforests (e.g. the Ambatovaky Special Reserve or the Vondrozo Forest Corridor) and extensive regions of arid western forests remain largely unexplored for their orchid diversity. Our sampling bias-corrected analysis also indicated that future inventories should be conducted primarily in two of Madagascar’s most famous protected areas, Tsaratanàna Strict Nature Reserve and Ranomafana National Park. These two conservation zones have historically been important areas for orchid sampling, but many of the collections are relatively old and may not reflect the current status of their orchid diversity.

There is still much to learn about the orchids of Madagascar and their conservation remains a neglected priority. Our study highlighted that a significant number of species have not been seen for a long time and may already be extinct, as more than 25% of the Malagasy orchids have not been observed in the last 50 years. Conversely, our dataset surprisingly shows that several species, predicted by Roberts and Jarić (2020) to be extinct have been collected more recently (i.e. *Jumelleaspathulata* collected in 2003, *Liparislongipetala* collected in 2012 or *Liparisparva* collected in 1997; see Suppl. material 2). It also highlights the importance of continued monitoring and survey efforts to accurately assess the conservation status of Malagasy orchids.

Recent publications have highlighted Madagascar as a top global priority for orchid conservation (e.g. Zizka et al. (2021), Wraith et al. (2020), Vitt et al. (2023)). However, to date, less than 25% of Malagasy orchids have been fully assessed by the IUCN, and the conservation status of most Malagasy orchids is currently unknown. To address this significant gap, we urge further research on the conservation status of orchids in Madagascar, as well as increased conservation efforts “on the ground” to protect these unique and threatened species. Efforts should also be made to engage local communities in these conservation efforts (Birkinshaw et al. 2013). Many Malagasy orchids are found in areas that are also important for agriculture and other livelihood activities and local people often have a deep understanding of these plants and their uses. By working with local communities, conservationists could better understand the ecological and cultural significance of orchids in Madagascar and develop more effective conservation strategies that offset human impacts.

## Supplementary Material

FF1F2871-3A7D-5CAC-9E1C-B2E2043CC61F 10.3897/BDJ.11.e106223.suppl1 Supplementary material 1 Checklist of Orchidaceae collected in Madagascar between 1816 and 2020 Data type Plant checklist Brief description For each species, we provided the total number of botanical records in our global dataset (see Supplementary material 2), the year of last collection, their distribution by country according to Govaert et al. (2022) and their published IUCN categories (IUCN 2022). File: oo_896658.xlsx https://binary.pensoft.net/file/896658 Droissart Vincent (IRD), Verlynde Simon (NYBG), Ramandimbisoa Brigitte (MBG), Andriamahefarivo Lalao (MBG) and Stévart Tariq (MBG)

B4707373-769D-5414-94FF-26274D1ED3D2 10.3897/BDJ.11.e106223.suppl2 Supplementary material 2 Global dataset of orchid specimens collected to date in Madagascar Data type Herbarium records Brief description We compiled and standardised a global dataset of orchid specimens collected between 1816 and 2020 in Madagascar using five public databases as main data sources: the Missouri Botanical Garden’s Tropicos database (http://www.tropicos.org/, last excerpted 10 March 2021), the GBIF database (GBIF 2019), the MNHN’s vascular plant database (https://science.mnhn.fr/institution/mnhn/collection/p/item/search), the BM database (https://data.nhm.ac.uk/search) and the Kew Herbcat database (http://apps.kew.org/herbcat/). We also reviewed the literature to add new or missing records (Hermans et al. 2017, Hermans et al. 2020a, Hermans et al. 2020b, Hermans et al. 2020c, Hermans et al. 2021a, Hermans et al. 2021b, c, d, Hermans et al. 2021e). Given the rarity of several listed species, we have not provided detailed GPS coordinates and have rounded these to the nearest 0.1 degrees. File: oo_896660.xlsx https://binary.pensoft.net/file/896660 Droissart Vincent (IRD), Verlynde Simon (NYBG), Ramandimbisoa Brigitte (MBG), Andriamahefarivo Lalao (MBG) and Stévart Tariq (MBG)

## References

[B9745613] Antonelli Alexandre, Smith Rhian J., Perrigo Allison L., Crottini Angelica, Hackel Jan, Testo Weston, Farooq Harith, Torres Jiménez Maria F., Andela Niels, Andermann Tobias, Andriamanohera Andotiana M., Andriambololonera Sylvie, Bachman Steven P., Bacon Christine D., Baker William J., Belluardo Francesco, Birkinshaw Chris, Borrell James S., Cable Stuart, Canales Nataly A., Carrillo Juan D., Clegg Rosie, Clubbe Colin, Cooke Robert S. C., Damasco Gabriel, Dhanda Sonia, Edler Daniel, Faurby Søren, de Lima Ferreira Paola, Fisher Brian L., Forest Félix, Gardiner Lauren M., Goodman Steven M., Grace Olwen M., Guedes Thaís B., Henniges Marie C., Hill Rowena, Lehmann Caroline E. R., Lowry Porter P., Marline Lovanomenjanahary, Matos-Maraví Pável, Moat Justin, Neves Beatriz, Nogueira Matheus G. C., Onstein Renske E., Papadopulos Alexander S. T., Perez-Escobar Oscar A., Phelps Leanne N., Phillipson Peter B., Pironon Samuel, Przelomska Natalia A. S., Rabarimanarivo Marina, Rabehevitra David, Raharimampionona Jeannie, Rajaonah Mamy Tiana, Rajaonary Fano, Rajaovelona Landy R., Rakotoarinivo Mijoro, Rakotoarisoa Amédée A., Rakotoarisoa Solofo E., Rakotomalala Herizo N., Rakotonasolo Franck, Ralaiveloarisoa Berthe A., Ramirez-Herranz Myriam, Randriamamonjy Jean Emmanuel N., Randriamboavonjy Tianjanahary, Randrianasolo Vonona, Rasolohery Andriambolantsoa, Ratsifandrihamanana Anitry N., Ravololomanana Noro, Razafiniary Velosoa, Razanajatovo Henintsoa, Razanatsoa Estelle, Rivers Malin, Sayol Ferran, Silvestro Daniele, Vorontsova Maria S., Walker Kim, Walker Barnaby E., Wilkin Paul, Williams Jenny, Ziegler Thomas, Zizka Alexander, Ralimanana Hélène (2022). Madagascar's extraordinary biodiversity: Evolution, distribution, and use. Science.

[B9745702] Birkinshaw Chris, Lowry II Porter P., Raharimampionona Jeannie, Aronson James (2013). Supporting Target 4 of the Global Strategy for Plant Conservation by Integrating Ecological Restoration into the Missouri Botanical Garden's Conservation Program in Madagascar. Annals of the Missouri Botanical Garden.

[B9745726] Callmander Martin W., Schatz George E., Lowry II Porter P., Laivao Michel O., Raharimampionona Jeannie, Andriambololonera Sylvie, Raminosoa Tantely, Consiglio Trisha K. (2007). Identification of priority areas for plant conservation in Madagascar using Red List criteria: rare and threatened Pandanaceae indicate sites in need of protection. Oryx.

[B9745711] Callmander Martin W., Phillipson Peter B., Schatz George E., Andriambololonera Sylvie, Rabarimanarivo Marina, Rakotonirina Nivo, Raharimampionona Jeannie, Chatelain Cyrille, Gautier Laurent, Lowry II Porter P. (2011). The endemic and non-endemic vascular flora of Madagascar updated. Plant Ecology and Evolution.

[B9745739] Chase M. W., Christenhusz M. J. M., Schuiteman A. (2020). Expansion of *Calanthe* to include the species of *Cephalantheropsis*, *Gastrorchis* and *Phaius* (Collabieae; Orchidaceae). Phytotaxa.

[B9745748] Chase M. W., Schuiteman A., Kumar P. (2021). Expansion of the orchid genus *Eulophia* (Eulophiinae; Epidendroideae) to include *Acrolophia*, *Cymbidiella*, *Eulophiella*, *Geodorum*, *Oeceoclades* and *Paralophia*. Phytotaxa.

[B9745757] Dauby G., Hardy O. J. (2012). Sampled-based estimation of diversity sensu stricto by transforming Hurlbert diversities into effective number of species. Ecography.

[B9745766] ESRI (2020). ArcGIS 10.8.1. for desktop..

[B9745774] GBIF (2019). GBIF Occurrence Download. https://www.gbif.org/occurrence/download/0002828-190320150433242.

[B9745782] Govaerts R., Bernet P., Kratochvil K., Gerlach G., Carr G., Pridgeon A. M., Alrich P., Pfahl J., Campaccil M. A., Baptista D. H., Tigges H., Shaw J., Cribb P. J., George A., Kreuz K., Wood J. (2022). World checklist of Orchidaceae. http://www.kew.org/wcsp/.

[B9745828] Hardy O. J. (2010). BiodivR 1.2. A program to compute statistically unbiased indices of species diversity within sample and species similarity between samples using rarefaction principles. http://ebe.ulb.ac.be/ebe/Software.html.

[B10427494] Hermans J., Hermans C., Du Puy D., Cribb P. J., Bosser J. (2007). Orchids of Madagascar.

[B9745848] Hermans Johan, Cribb Phillip (2014). New species and new names in Malagasy orchids (Orchidaceae). Kew Bulletin.

[B9745836] Hermans Johan, Andriantiana Jacky L., Sieder Anton, Kiehn Michael, Cribb Phillip, Rajavelona Landy, Gardiner Lauren M. (2017). New species and nomenclatural changes in *Cynorkis* (Orchidaceae: Orchidoideae) from Madagascar and the Mascarenes. Kew Bulletin.

[B10427435] Hermans Johan (2020). Cynorkis prehsleri, a new orchid from northern Madagascar. Curtis's Botanical Magazine.

[B10427448] Hermans Johan (2020). Gastrodia agnicellus, a new holomycotrophic orchid from southeast Madagascar. Curtis's Botanical Magazine.

[B9745927] Hermans Johan, Verlynde Simon, Rajaovelona Landy, Cribb Phillip J., Hervouet Jean-Michel (2020). New species and nomenclatural changes in *Angraecum* (Orchidaceae) from Madagascar. Kew Bulletin.

[B9745896] Hermans Johan, Rajaovelona Landy, Cribb Phillip, Hervouet Jean-Michel, Sieder Anton, Andriantiana Jacky (2020). New species and nomenclatural changes in *Cynorkis* (Orchidaceae) from Madagascar, the Comoros and the Mascarenes. Kew Bulletin.

[B9745916] Hermans Johan, Verlynde Simon, Cribb Phillip, Ramandimbisoa Brigitte, Hervouet Jean-Michel, Bernet Patrice (2020). Malaxideae (Orchidaceae) in Madagascar, the Mascarenes, Seychelles and Comoro Islands. Kew Bulletin.

[B10427457] Hermans Johan, Cribb Phillip (2021). New combinations and other taxonomic changes for the forthcoming ‘Flore des Mascareignes’ Orchidaceae accounts. Lankesteriana.

[B9745869] Hermans Johan, Rajaovelona Landy, Cribb Phillip (2021). New species in Orchidaceae from Madagascar. Kew Bulletin.

[B9745878] Hermans Johan, Rajaovelona Landy, Cribb Phillip (2021). *Bulbophyllumlanterna*, a new species in Dendrobiinae (Orchidaceae) from Madagascar. Kew Bulletin.

[B9745887] Hermans Johan, Rajaovelona Landy, Cribb Phillip (2021). *Angraecuminflatum*, a new species in Angraecinae (Orchidaceae) from Madagascar. Kew Bulletin.

[B9745907] Hermans J., Sieder Anton, Rajaovelona Landy, Andriantiana Jacky (2021). *Angraecumidae*, a new orchid from Madagascar. Orchids.

[B9745857] Hermans Johan, Gamisch Alexander, Rajaovelona Landy, Fischer Gunter A., Cribb Phillip, Sieder Anton, Andriantiana Jacky (2021). New species and nomenclatural changes in *Bulbophyllum* (Orchidaceae) from Madagascar. Kew Bulletin.

[B10427504] Hermans J., Rajaovelona L., Goodman S. M. (2022). The Natural History of Madagascar, volume 1, p. 559-567.

[B10427475] Hillerman F. E., Holst A. W. (1986). An Introduction to the Cultivated Angraecoid Orchids of Madagascar.

[B9745937] Hsieh T. C., Ma K. H., Chao Anne (2016). iNEXT: an R package for rarefaction and extrapolation of species diversity (Hill numbers). Methods in Ecology and Evolution.

[B9745946] Humphreys Aelys M., Govaerts Rafaël, Ficinski Sarah Z., Nic Lughadha Eimear, Vorontsova Maria S. (2019). Global dataset shows geography and life form predict modern plant extinction and rediscovery. Nature Ecology & Evolution.

[B9745956] IPNI (2022). International Plant Names Index. Published on the Internet by the Royal Botanic Gardens, Kew, Harvard University Herbaria & Libraries and Australian National Botanic Gardens. http://www.ipni.org.

[B9745964] IUCN (2022). The IUCN Red List of Threatened Species. Version 2022-1.. http://www.iucnredlist.org.

[B9745972] Kier Gerold, Kreft Holger, Lee Tien Ming, Jetz Walter, Ibisch Pierre L., Nowicki Christoph, Mutke Jens, Barthlott Wilhelm (2009). A global assessment of endemism and species richness across island and mainland regions. Proceedings of the National Academy of Sciences.

[B10427521] Mackay-Smith Thomas H., Roberts David L. (2019). Accuracy in the identification of orchids of the genus Angraecum by taxonomists and non-taxonomists. Kew Bulletin.

[B9746000] Catalogue Madagascar (2022). Catalogue of the Plants of Madagascar. http://www.tropicos.org/Project/Madagascar.

[B9746008] Rakotoarinivo M., Dransfield J., Bachman S. P., Moat J., Baker W. J. (2014). Comprehensive Red List assessment reveals exceptionally high extinction risk to Madagascar palms. PLOS One.

[B9746026] Roberts David L., Jarić Ivan (2020). Inferring the extinction of species known only from a single specimen. Oryx.

[B9746035] Soulebeau Anaëlle, Pellens Roseli, Lowry Porter P., Aubriot Xavier, Evans Margaret E. K., Haevermans Thomas, Pellens Roseli, Grandcolas Philippe (2016). Biodiversity Conservation and Phylogenetic Systematics: Preserving our evolutionary heritage in an extinction crisis.

[B9746050] Thiers B. (2018, continuously updated). Index Herbariorum: A global directory of public herbaria and associated staff. http://sweetgum.nybg.org/ih/.

[B9746058] Vieilledent Ghislain, Grinand Clovis, Rakotomalala Fety A., Ranaivosoa Rija, Rakotoarijaona Jean-Roger, Allnutt Thomas F., Achard Frédéric (2018). Combining global tree cover loss data with historical national forest cover maps to look at six decades of deforestation and forest fragmentation in Madagascar. Biological Conservation.

[B9754534] Vitt Pati, Taylor Amanda, Rakosy Demetra, Kreft Holger, Meyer Abby, Weigelt Patrick, Knight Tiffany M. (2023). Global conservation prioritization for the Orchidaceae. Scientific Reports.

[B9746070] WCVP (2022). World Checklist of Vascular Plants, version 2.0. Facilitated by the Royal Botanic Gardens, Kew. Published on the Internet. http://wcvp.science.kew.org/.

[B9746078] Whittaker R. J., Fernández-Palacios J. M. (2007). Island biogeography: ecology, evolution, and conservation.

[B9746086] Wraith Jenna, Norman Patrick, Pickering Catherine (2020). Orchid conservation and research: An analysis of gaps and priorities for globally Red Listed species. Ambio.

[B9746095] Zizka Alexander, Silvestro Daniele, Vitt Pati, Knight Tiffany M. (2021). Automated conservation assessment of the orchid family with deep learning. Conservation Biology.

